# NSP-Dependent Simple Nitrile Formation Dominates upon Breakdown of Major Aliphatic Glucosinolates in Roots, Seeds, and Seedlings of *Arabidopsis thaliana* Columbia-0

**DOI:** 10.3389/fpls.2016.01821

**Published:** 2016-12-01

**Authors:** Ute Wittstock, Kathrin Meier, Friederike Dörr, Beena M. Ravindran

**Affiliations:** Institute of Pharmaceutical Biology, Technische Universität BraunschweigBraunschweig, Germany

**Keywords:** glucosinolate breakdown, myrosinase, nitrile-specifier protein, simple nitrile, organ-specificity

## Abstract

One of the best-studied plant defense systems, the glucosinolate-myrosinase system of the Brassicales, is composed of thioglucosides known as glucosinolates and their hydrolytic enzymes, the myrosinases. Tissue disruption brings these components together, and bioactive products are formed as a consequence of myrosinase-catalyzed glucosinolate hydrolysis. Among these products, isothiocyanates have attracted most interest as chemical plant defenses against herbivores and pathogens and health-promoting compounds in the human diet. Previous research has identified specifier proteins whose presence results in the formation of alternative product types, e.g., nitriles, at the expense of isothiocyanates. The biological roles of specifier proteins and alternative breakdown products are poorly understood. Here, we assessed glucosinolate breakdown product profiles obtained upon maceration of roots, seedlings and seeds of *Arabidopsis thaliana* Columbia-0. We identified simple nitriles as the predominant breakdown products of the major endogenous aliphatic glucosinolates in root, seed, and seedling homogenates. In agreement with this finding, genes encoding nitrile-specifier proteins (NSPs) are expressed in roots, seeds, and seedlings. Analysis of glucosinolate breakdown in mutants with T-DNA insertions in any of the five *NSP* genes demonstrated, that simple nitrile formation upon tissue disruption depended almost entirely on *NSP2* in seeds and mainly on *NSP1* in seedlings. In roots, about 70–80% of the nitrile-forming activity was due to *NSP1* and *NSP3*. Thus, glucosinolate breakdown product profiles are organ-specifically regulated in *A. thaliana* Col-0, and high proportions of simple nitriles are formed in some parts of the plant. This should be considered in future studies on biological roles of the glucosinolate-myrosinase system.

## Introduction

**FIGURE 1:**
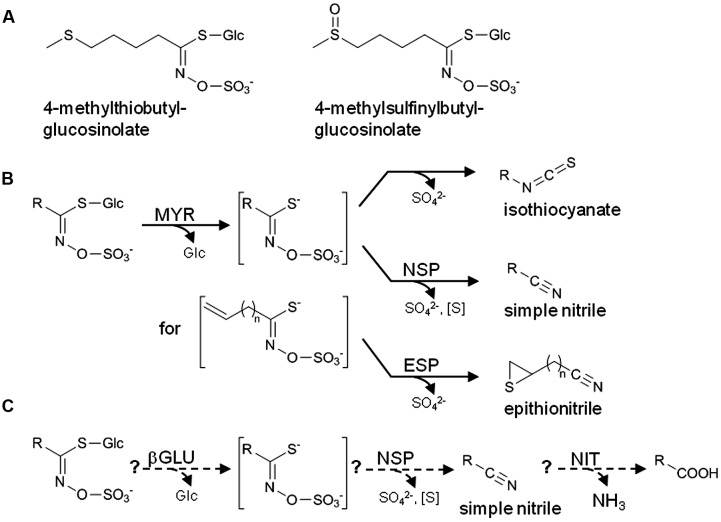
**The glucosinolate-myrosinase system.** **(A)** Structures of the two major aliphatic glucosinolates of *A. thaliana* Col-0. **(B)** Scheme of glucosinolate breakdown upon tissue disruption. In the absence of specifier proteins, isothiocyanates are formed by spontaneous rearrangement of the glucosinolate aglucone. In the presence of specifier proteins, simple nitriles and epithionitriles are formed instead of isothiocyanates. Formation of epithionitriles requires a terminal double bond in the aglucone side chain. **(C)** Scheme of the hypothetical pathway of glucosinolate turnover in intact tissue. Formation of potentially harmful isothiocyanates is prevented, and glucosinolates are used as a source of nutrients. βGLU, β-glucosidase; NSP, nitrile-specifier protein; ESP, epithiospecifier protein; NIT, nitrilase; MYR, myrosinase; R, variable side chain. Water molecules involved in the hydrolytic reactions (MYR, βGLU, NIT) are omitted for clarity.

*Arabidopsis thaliana* (Brassicaceae) lends itself as a model to study biological roles of glucosinolates, a group of amino acid-derived thioglucosides present in the Brassicales (**Figure 1A**) (Halkier and Gershenzon, 2006; Kopriva, 2016), due to the wealth of information on glucosinolate metabolism in this species and the relative ease with which its metabolism can be manipulated. Numerous studies have used *A. thaliana* mutants with defects in glucosinolate biosynthesis or ectopic glucosinolate production to address defensive roles of different glucosinolate classes, i.e., glucosinolates derived from aliphatic amino acids, Phe/Tyr, or Trp (Brader et al., 2006; Beekweelder et al., 2008; Kim et al., 2008; Schlaeppi et al., 2008; Kissen et al., 2009a; Müller et al., 2010; Falk et al., 2014). Glucosinolates are components of an activated plant defense triggered by tissue disruption. Thus, the actual defense compounds are formed upon hydrolysis of glucosinolates by thioglucosidases termed myrosinases (EC 3.2.1.147) which are released from separate storage compartments upon cellular disintegration (“mustard oil bomb”) (Matile, 1980; Halkier and Gershenzon, 2006; Kissen et al., 2009b) (**Figure 1B**). The best studied products of glucosinolate breakdown, the isothiocyanates (or mustard oils), are highly reactive, irritant and toxic to a wide range of organisms (Wittstock et al., 2003). Thus, defensive roles of glucosinolates are likely associated with the corresponding isothiocyanates, and this has also been demonstrated in several studies (Lambrix et al., 2001; Agrawal and Kurashige, 2003; Burow et al., 2006b; Stotz et al., 2011). However, depending on the plant species, organ and the glucosinolate structure, other products such as simple nitriles, epithionitriles, and organic thiocyanates can also be formed upon glucosinolate hydrolysis, and the biological roles of these products are much less understood (Wittstock and Burow, 2007). In *A. thaliana* and many other species of the Brassicaceae, formation of non-isothiocyanate products depends on so-called specifier proteins, namely epithiospecifier protein (ESP) and nitrile-specifier proteins (NSPs), as well as on additional factors such as the epithiospecifier modifier gene (*ESM1*) (Lambrix et al., 2001; Zhang et al., 2006; Burow et al., 2009; Kissen and Bones, 2009; Kuchernig et al., 2012). Specifier proteins do not act on glucosinolates, but are assumed to convert the glucosinolate aglucones released by myrosinases specifically into non-isothiocyanate products (Wittstock and Burow, 2007). For example, presence of ESPs leads to formation of epithionitriles upon myrosinase-catalyzed hydrolysis of alkenyl glucosinolates while NSPs promote simple nitrile formation upon hydrolysis of all structural types of glucosinolates (Wittstock and Burow, 2010) (**Figure 1B**). However, it can be difficult to discriminate between contributions of ESP and NSP, as simple nitriles of non-alkenyl glucosinolates can also be generated by ESP (Burow et al., 2006a,b).

Natural variation in both glucosinolate biosynthesis and breakdown genes generates an astonishing structural diversity of product profiles upon activation in different accessions of *A. thaliana* (Lambrix et al., 2001; Zhang et al., 2006; Wentzell and Kliebenstein, 2008; Witzel et al., 2013, 2015). Within a single accession, glucosinolate profiles are developmentally and organ-specifically regulated and respond to biotic and abiotic stresses (Harada et al., 2000; Brader et al., 2001; Petersen et al., 2002; Brown et al., 2003; Witzel et al., 2013). Studies in *A. thaliana* and other species of the Brassicaceae suggest that breakdown product types are also determined organ-specifically and in response to herbivores and pathogens (Burow et al., 2007a,b; Wentzell and Kliebenstein, 2008; Burow et al., 2009; Kuchernig et al., 2011; Witzel et al., 2015). However, breakdown product profiles in organs other than leaves have rarely been investigated (Falk et al., 2014; Witzel et al., 2015). When external allylglucosinolate is added to homogenates of *A. thaliana* Columbia-0 (Col-0), the isothiocyanate accounts for about 95% of its breakdown products in homogenates of rosettes, cauline leaves and flowers, but for only 10–20% in seedlings and roots which produce mainly the simple nitrile (Wentzell and Kliebenstein, 2008).

The Col-0 accession of *A. thaliana* is widely used for genetic screens and in bioassays with herbivores and pathogens due to the availability of mutant collections. Besides indolic glucosinolates, Col-0 plants accumulate methylthio- and methylsulfinylalkylglucosinolates in most organs (**Figure 1A**) and hydroxy- and benzoyloxyalkylglucosinolates in seeds (Petersen et al., 2002; Brown et al., 2003). Homogenates of rosette leaves contain mainly isothiocyanates, but also simple nitriles as products of glucosinolate breakdown (Lambrix et al., 2001; Burow et al., 2009). Due to an insertion upstream of the ESP coding sequence, Col-0 is a natural knockout of ESP and ESP activity is not detectable in leaf extracts (Lambrix et al., 2001; Burow et al., 2007b). However, Col-0 possesses five functional *NSP* genes, *NSP1–NSP5*, whose biological roles are only poorly understood (Burow et al., 2009; Kissen and Bones, 2009). Based on microarray data, *NSP1–NSP5* are differentially expressed in different organs and developmental stages (Kissen and Bones, 2009) (Supplementary Figure 1). *NSP1*, *NSP3*, and *NSP4* are tandem genes represented by one probe on the Affymetrix ATH1 microarray due to their high nucleotide sequence identity. Thus, the *NSP1*/*NSP3*/*NSP4* probe does not differentiate between these genes. It detects high expression levels in seedlings and roots and intermediate levels in rosette leaves. *NSP2* appears to be highly expressed in siliques and at low levels in roots and rosettes while *NSP5* is expressed at intermediate levels throughout the plant at the vegetative and generative stage based on microarray data. However, the contribution of each of the genes to glucosinolate breakdown *in vivo* has not been established and it has remained unclear if redundant genes are involved in glucosinolate breakdown in different organs and developmental stages. As an exception, an *NSP1* T-DNA insertion line (*nsp1-1*) has been demonstrated to be deficient in constitutive and herbivore-induced simple nitrile formation in rosette leaves (Burow et al., 2009). While this indicates that *NSP1* is the major *NSP* of rosettes *in vivo*, it raises questions about possible roles and post-transcriptional regulation of the other *NSP* genes some of which also seem to be transcribed in rosettes.

The goal of this study was to clarify which types of breakdown products are formed from endogenous glucosinolates upon disruption of *A. thaliana* Col-0 roots, seeds, and seedlings and to evaluate the contribution of each individual *NSP* gene to breakdown product profiles through analysis of T-DNA mutants.

## Materials and Methods

### Plant Growth and Harvest

*Arabidopsis thaliana* (L.) HEYH. plants of the Col-0 accession [wild-type (WT) and homozygous mutants in parallel] were grown on soil or in aeroponic culture on Seramis clay granules (Seramis, Mogendorf, Germany; Vaughan et al., 2011) in a controlled environment chamber at 22°C, 60–70% relative humidity, and 300 μmol m^-2^ s^-1^ photosynthetically active radiation with a photoperiod of 10 h. To obtain seeds, plants were transferred to long-day conditions (photoperiod of 16 h) 2–3 weeks after sowing. Soil was composed of 80% Compo germination potting compost (Münster, Germany), 10% sand, 10% Perligran (Knauf Perlite, Germany) and supplemented with 2 g/l Triabon (Compo) and 1 g/l Sierrablen (Scotts, Heerlen, Netherlands) fertilizer. For aeroponic growth, 2-week-old plants were transferred from soil to pots (5 cm diameter) filled with Seramis clay granules which had been soaked in Hoagland’s solution (Gibeaut et al., 1997). Pots were placed in trays containing Hoagland’s solution. Glucosinolate content and composition of roots, seedlings and seeds of Col-0 plants corresponded largely with previously reported values (Supplementary Figures 2 and 3) (Petersen et al., 2002; Brown et al., 2003). Seeds of mutant lines (Supplementary Table 1) were obtained from the European Arabidopsis Stock Centre, Nottingham, (NASC) and germinated on Murashige and Skoog medium containing 0.9% (w/v) agar and 50 μg/ml kanamycin before transfer to soil. T-DNA insertions and homozygosity were confirmed by PCR (see Verification of Mutant Alleles). Homozygous lines were grown on soil. Plant ages are given from the date of imbibition. Eight-day-old seedlings had two true leaves.

### Semiquantitative RT-PCR

With the exception of seeds, which were used after 1–3 months of storage at room temperature in the dark, plant material was frozen in liquid nitrogen and used after storage at -80°C. Total RNA was isolated by the lithium chloride method according to Raha et al. (1990). Ribolock RNase Inhibitor (Thermo Scientific) was added at 1.3 u/μl before DNase I (Thermo Scientific) treatment according to the manufacturer’s instructions. Quality and quantity of RNA were evaluated spectrophotometrically and by gel electrophoresis on 1.5% (w/v) native agarose gels. For expression analysis in different organs of WT plants, first-strand cDNA synthesis was performed on at least three different amounts of RNA (0.25, 0.1, or 0.05 μg) to ensure data points within the linear range using Revert AidH Minus RT (Thermo Scientific) according to the manufacturer’s instructions. PCR with gene-specific primers (10 pmol each; Supplementary Table 2) was performed in a total volume of 25 μl using 1 μl RT reaction. PCR reactions were done with 1.25 u DreamTaq (Thermo Scientific) in 1x DreamTaq buffer supplemented with 200 μM dNTPs (Fermentas) in a Biometra TProfessional Basic Gradient thermocycler with the following temperature program: 94°C for 2 min; 30 cycles (*NSP2*, *NSP5*, *Actin8*) or 34 cycles (*NSP1*, *NSP3*, *NSP4*) of 94°C for 30 s, annealing temperature (Supplementary Table 2) for 1 min, 72°C for 1 min; final extension at 72°C for 10 min. For WT–mutant comparisons, RT reactions were set up with 0.25 μg RNA, and amounts of cDNA corresponding to 0.025–1 μl RT reaction (depending on organ and gene) were subjected to PCR as described above. PCR products were analyzed by electrophoresis on 1–2% (w/v) agarose gels and visualized by Midori Green Advance DNA Stain (Nippon Genetics Europe). For each organ/stage, identity of the PCR products was confirmed by sequencing. At least two experiments with plant material from independently grown sets of plants were done, and within each experiment cDNA synthesis and RT-PCR were repeated several times with similar results.

### qPCR

RNA samples were generated and evaluated as described in Section “Semiquantitative RT-PCR.” Roots and rosette leaves, respectively, from three individuals were pooled before RNA isolation. RNA (1 μg) was used for cDNA synthesis with Revert AidH Minus RT (Thermo Scientific) and oligo-(dT)-primer according to the manufacturer’s instructions. PCRs were setup with three technical replicates in a total volume of 10 μl containing 5 μl iTaq Universal SYBR Green Supermix (Bio-Rad), 10 pmol of each primer, and 3 μl cDNA dilution (1:1000) and run on a Bio-Rad CFX Connect PCR Detection System with the following temperature program: 95°C for 30 s, 40 cycles of 95°C for 5 s, and 60°C for 30 s. Melting curves were recorded from 65 to 95°C with increments of 0.5°C for 5 s. Primer efficiencies (E) were determined with serial dilutions (10x–100.000x diluted) of cDNA and three technical replicates. Mean values for 1+E were in the range of 1.93 and 2.04 with relative standard deviations (SD) of <5% (Supplementary Table 3). CT values were calculated as means of three technical replicates. To compare expression levels in different organs, the CT values of *NSP1–NSP5* were normalized to the CT values of two reference genes (Ref), *elongation factor 1α* (*EF1α*) and *ubiquitin 10* (*UBI10*), to obtain ΔCT values. The expression level of the gene of interest (GOI) in each plant sample was expressed as (1+E)^-ΔCT^ = (1+E_Ref_)^CTRef^/(1+E_GOI_)^CTGOI^, i.e., by a variant of the 2^-ΔCT^ method with inclusion of primer efficiencies (Livak and Schmittgen, 2001). The relative quantification model was used to compare expression levels in mutants vs. WT. CT values for *NSP1*–*NSP5* or the reference genes *EF1α*, *UBI10*, or β*-tubulin 6* (*TUB6*) in mutant samples were subtracted from those of WT samples to obtain ΔCTGOI or ΔCTRef, respectively. Relative expression (mutant vs. WT) was expressed as (1 + E_GOI_)^-ΔCTGOI(WT-mutant)^/(1 + E_Ref_)^-ΔCTRef(WT-mutant)^. Biological replicates were from three independently grown sets of plants. To ensure specificity, water controls were included, identity of PCR products was confirmed for each primer pair by cloning and sequencing from seed (*NSP2*) or root samples (*NSP1*, *NSP3*–*NSP5*, reference genes), and melting curves were checked for every run.

### Verification of Mutant Alleles

PCR was conducted with 1.25 units DreamTaq-Polymerase (Fermentas) in a total volume of 25 μl Dream Taq buffer (Fermentas, with 2 mM MgCl_2_) supplemented with 200 μM dNTPs, and 20 pmol of each primer using 1 μg genomic DNA isolated from leaves and the following temperature program: 2 min at 94°C; 33 cycles of 94°C for 45 s, 57°C for 45 s, 72°C for 60 s; final extension at 72°C for 10 min. Primer combinations are listed in Supplementary Table 1 and Primer sequences in Supplementary Table 4. PCR reactions were analyzed on 1.5% agarose gels containing Midori Green Advance DNA Stain (Nippon Genetics Europe) for visualization. The identity of PCR products was confirmed by sequencing.

### Glucosinolate Hydrolysis Product Analysis in Tissue Homogenates

Freshly harvested rosettes (300–400 mg) and roots (100–400 mg) of individual plants or batches of 300–400 mg seedlings were ground with sea sand using mortar and pestle in 50 mM MES buffer, pH 6.0 (100 μl buffer per 100 mg plant material). After 5–10 min incubation at room temperature, plant parts were removed by centrifugation, and 20–30 μl internal standard (phenylcyanide, 100 ng μl^-1^ in MeOH) were added to the supernatant. Extracts from seeds (batches of 30–40 mg, after up to 3 months of storage at room temperature in the dark) were made in the same way, but 100 μl buffer was used per 10 mg plant material and internal standard was used at 30–60 μl per sample. The supernatants were extracted twice with 750 μl dichloromethane (roots) or 1.5 ml dichloromethane (seeds, seedlings). Organic phases were dried over Na_2_SO_4_, concentrated in an air stream to about 200 μl and analyzed by GC-MS and GC-FID using an Agilent 6890N series gas chromatograph with an HP5MS column (30 m × 0.25 mm × 0.25 μm; Wicom, Heppenheim, Germany), splitless injection at 200°C (injection volume 1 μl), and the following temperature program: 35°C for 3 min, 12°C min^-1^ to 280°C, 30°C min^-1^ to 300°C (MS) or to 310°C (FID), 3 min final hold. MS and FID were carried out as described previously (Lambrix et al., 2001). Products were identified by comparison of mass spectra and retention times with those of authentic standards and with published MS spectra (Spencer and Daxenbichler, 1980). FID peaks for which retention time-based assignment was possible were quantified based on FID peak area and response factors calculated based on effective carbon numbers (ECN) (Scanlon and Willis, 1985; Lambrix et al., 2001).

### Glucosinolate Analysis

Glucosinolate profiles were determined by HPLC of the corresponding desulfoglucosinolates (Thies, 1979). Freeze-dried plant material (10–20 mg roots, 20–30 mg seedlings) or 20 mg seeds were incubated with 400–500 μl methanol at 94°C for 5 min followed by addition of 400–500 μl water, 50 μl 1 mM 4-hydroxybenzylglucosinolate (internal standard) and three glass beads (steel beads for seed samples). Samples were transferred to a paint shaker for 90 s and to an ultrasonic bath for 4 min. Insoluble material was pelleted and washed once with 400 μl water. Supernatants were pooled and desulfoglucosinolates prepared according to (Burow et al., 2006b). HPLC analysis was done on an Agilent HP1200 Series instrument equipped with a LiChrospher RP18 column (250 mm × 4.6 mm, 5 μm particle size; Wicom) using a water (solvent A)-acetonitrile (solvent B) gradient at a flow rate of 1 ml min^-1^ at 25°C (injection volume 25 μl). The gradient was as follows: 1.5% B (1 min), 1.5–5% B (5 min), 5–7% B (2 min), 7–21% (10 min), 21–29% (5 min), 29–43% (7 min), 43–93% (5 min), 93% B (5 min), 93–1.5% B (5 min), and 1.5% B (5 min). The eluent was monitored by diode array detection between 190 and 360 nm (2 nm interval). Desulfoglucosinolates were identified based on comparison of retention times and UV absorption spectra with those of known standards (Reichelt et al., 2002) and quantified based on peak areas at 229 nm relative to the peak area of the internal standard (relative response factor 2.0 for aliphatic glucosinolates and 0.5 for indole glucosinolates (Burow et al., 2006b; Sarosh et al., 2010).

### Fresh-Weight/Dry Weight Comparison

Before hydrolysis product analysis, fresh weight of complete roots of individual aeroponically grown plants (6-week-old) was determined. Fresh roots had a mean mass (±SD) of 284 ± 106 mg (*N* = 16 from six experiments). Complete roots of individual aeroponically grown plants (6-week-old) grown in parallel were freeze-dried (for glucosinolate analysis) and their dry weight determined. Dried roots had a mean mass (±SD) of 15.7 ± 4.6 mg (*N* = 17, seven experiments). For 8-day-old soil-grown seedlings, fresh weight (fw) was determined per batch directly after harvest. Dry weight (dw) of the same batches was determined after freeze-drying. The ratio fw/dw was 18.6 ± 0.6 (*N* = 6).

### Statistics

Statistical analyses were done with OriginPro 8. Homogeneity of variance was assessed using ANOVA with Levene’s Test (absolute and squared deviations) and the Brown–Forsythe Test. Unless otherwise stated, significant differences were identified in pairwise comparisons between WT and each mutant using Two-Sample *t*-Test for normally distributed data and Mann–Whitney *U* Test for not-normally distributed data.

## Results

**FIGURE 2:**
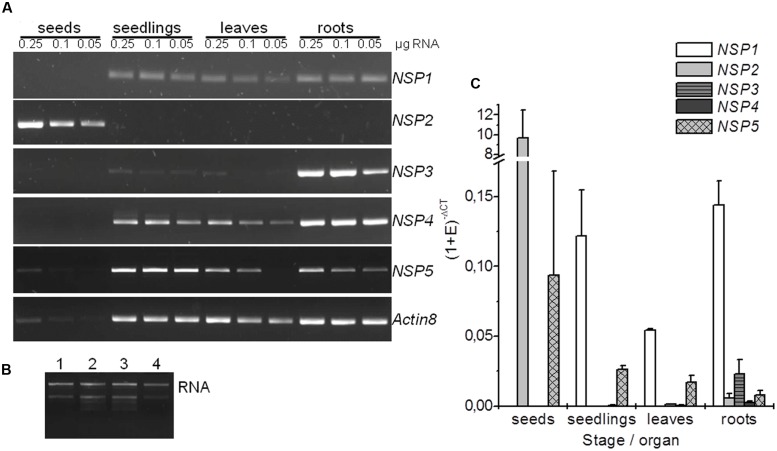
**Expression analysis of *NSP1–NSP5* in *A. thaliana* Col-0.** Results are shown for seeds, 8-day-old, soil-grown seedlings, as well as rosettes and roots of 6-week-old, aeroponically grown plants. **(A)** Total RNA (0.25, 0.1, and 0.05 μg) was used for cDNA synthesis and 1 μl RT reaction was subjected to PCR with the gene-specific primers indicated at the right. PCR reactions were analyzed by gel electrophoresis (2 μl per lane). Gel images are from one experiment that was repeated once with an independently grown set of plants and gave the same principal results. **(B)** Aliquots of RNA preparations used for cDNA synthesis were analyzed by gel electrophoresis on native agarose gels (0.25 μg total RNA per lane). A representative image is shown (1: seeds, 2: seedlings, 3: rosettes, 4: roots). **(C)** qPCR analysis using SYBR Green for quantification. The CT values of *NSP1*–*NSP5* were normalized to *EF1α* as reference (Ref) gene. The expression level of the gene of interest (GOI) in each tissue was expressed as (1 + E)^-ΔCT^ = (1 + E_Ref_)^CTRef^/(1 + E_GOI_)^CTGOI^ (E, primer efficiency). Means ± SD of *N* = 3 independently grown sets of plants.

In order to analyze expression of the *NSP1–NSP5* genes in *Arabidopsis*, we performed semiquantitative RT-PCR using gene-specific primers and RNA isolated from seeds, 8-day-old seedlings as well as rosettes and roots of 6-week-old aeroponically grown plants. We found differential expression of the *NSP* genes, including the tandem genes *NSP1*, *NSP3*, and *NSP4* (**Figures 2A,B**). *NSP2* expression was restricted to seeds. Of the tandem genes, *NSP3* was expressed almost exclusively in roots while *NSP4* was mostly expressed in roots, but well detectable in seedlings and rosettes. *NSP1* and *NSP5* expression was detected in seedlings, leaves and roots with slightly lower levels in leaves than in the other samples. *NSP5* transcript was also detectable at very low levels in seeds. For an intergenic comparison, we performed quantitative real-time PCR (qPCR) using *EF1α* (**Figure 2C**) and *UBI10* (Supplementary Figure 4) as reference genes. This largely confirmed the results of the semiquantitative study. Specifically, qPCR revealed a 10- to 100-fold higher expression level of *NSP2* in seeds than of any other *NSP* gene in any of the studied organs/stages (**Figure 2C**, Supplementary Figure 4). Of the tandem genes, *NSP1* had the by far highest expression levels regardless of the organ/stage analyzed while *NSP4* was expressed at 70- to 100-fold lower levels and *NSP3* expression proved to be largely root-specific (**Figure 2C**; Supplementary Figure 4). *NSP3* expression in roots and *NSP5* expression in seedlings and leaves reached about 10–15% and 20%, respectively, of the *NSP1* expression level (**Figure 2C**; Supplementary Figure 4). Based on these results, we expected that *NSP2* determines glucosinolate breakdown product profiles in seed homogenates, while mostly *NSP1* and *NSP3* contribute to simple nitrile formation in roots. The data further suggested that simple nitrile formation in seedlings and rosettes depends mostly on *NSP1* and *NSP5*. However, leaf homogenates of the *nsp1-1* mutant lacked simple nitriles (Burow et al., 2009). This identified NSP1 as the major NSP of rosette leaves despite *NSP5* expression. In order to expand this type of analysis to other organs and *NSP* genes, we analyzed glucosinolates and glucosinolate breakdown products in roots, seedlings, and seeds of T-DNA insertion mutants of each *NSP* gene.

**FIGURE 3:**
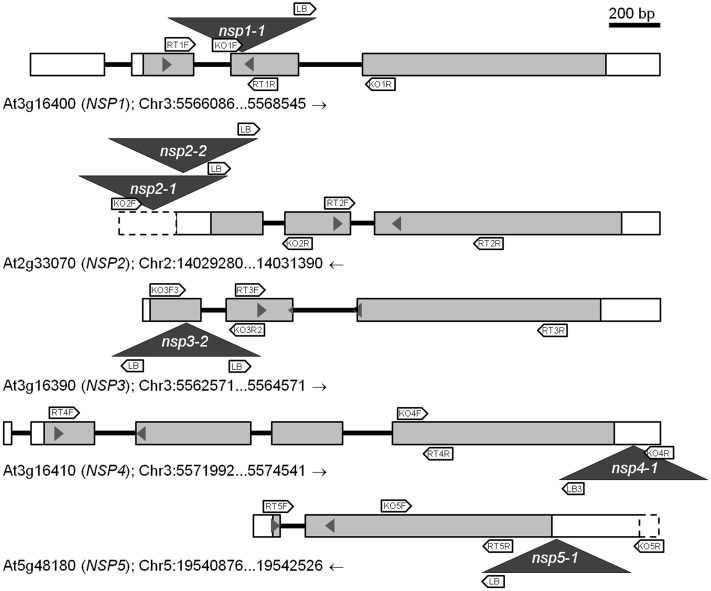
**Scheme of wild-type (WT) and mutant alleles of *NSP1–NSP5*.** Exons are represented by boxes and introns by black bars. Gray filling indicates open reading frames and dashed boxes upstream and downstream sequence. T-DNA insertions are indicated by dark gray triangles. Block arrows indicate the approximate position of primers used to verify T-DNA insertions (KO; Supplementary Table 4) and in semiquantitative RT-PCR (RT; Supplementary Table 2). Small pairs of triangles within the boxes indicate the approximate position of primers used to analyze transcript levels by qPCR (Supplementary Table 3). Genome sections are labeled with AGI code (gene name), chromosome (Chr) number, and nucleotide numbers according to the genomic sequences at TAIR (the arrow indicates if forward (→) or reverse (←) sequence is shown). A scale bar is given at the top.

**FIGURE 4:**
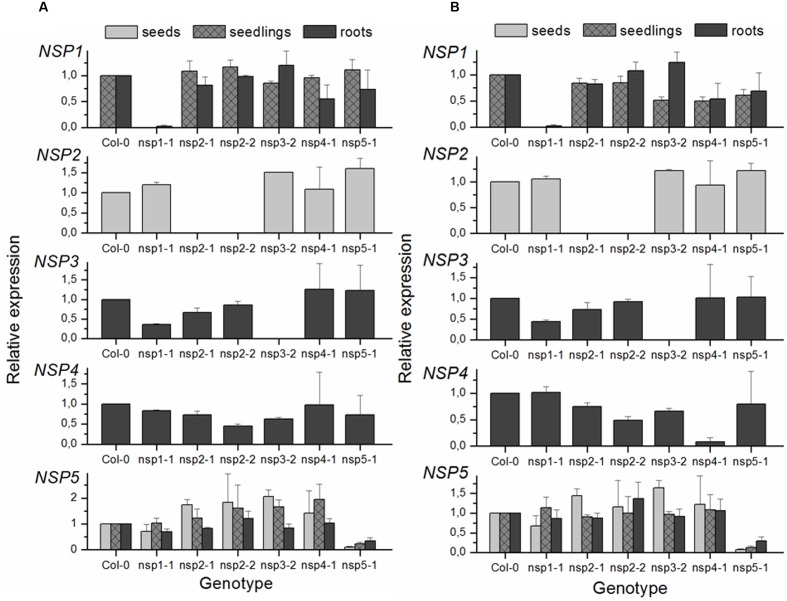
**qPCR analysis of *NSP1–NSP5* expression in T-DNA insertion lines of *A. thaliana* relative to Col-0 WT.** Gene expression was analyzed in seeds, 8-day-old, soil-grown seedlings, and roots of 6-week-old, aeroponically grown plants. SYBR Green was used for quantification. Relative expression (mutant vs. WT) was calculated as (1 + E_GOI_)^-ΔCTGOI(WT-mutant)^/(1 + E_Ref_)^-ΔCTRef(WT-mutant)^. **(A)** CT values of *NSP1*–*NSP5* were normalized to *EF1α* as reference (Ref) gene. **(B)** CT values of seed samples and CT values for *NSP2* were normalized to *UBI10*, those of all other samples and *NSP* genes to *TUB6*. Means ± SEM of *N* = 3 independently grown sets of plants.

Mutants were obtained from NASC and the position and orientation of T-DNA insertions (**Figure 3**) confirmed by PCR (Supplementary Table 1). The effect of the mutations on transcription of the *NSP1–NSP5* genes in seeds, seedlings and roots was analyzed by semiquantitative RT-PCR (Supplementary Figure 5) as well as by qPCR (**Figure 4**). Based on this, *nsp1-1*, *nsp2-1*, *nsp2-2*, and *nsp3-2* appeared to be knockout mutants while *nsp4-1* and *nsp5-1* still expressed *NSP4* and *NSP5*, respectively, but at reduced levels (**Figure 4**). The mutant alleles also affected expression of other *NSP* genes. A T-DNA insertion in one of the tandem genes (*nsp1-1*, *nsp3-2*, *nsp4-1*; **Figure 4**) usually resulted in reduced expression of at least one other tandem gene. Furthermore, T-DNA insertions in *NSP2*–*NSP4* were associated with increased *NSP5* expression, especially in seeds and seedlings (**Figure 4**). Taken together, T-DNA insertions strongly reduced or abolished target gene expression and had varying effects on expression of other *NSP* genes, including impairment of tandem gene expression and upregulation of *NSP5*.

**FIGURE 5:**
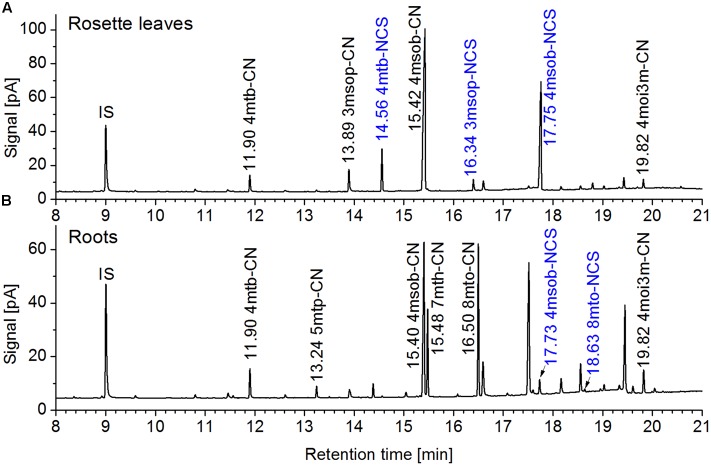
**Glucosinolate breakdown product profiles in homogenates of rosette leaves and roots of a 6-week-old aeroponically grown *A. thaliana* Col-0 plant.** Rosette leaves **(A)** and roots **(B)** of the same plant were homogenized in aqueous buffer and dichloromethane extracts of the homogenates were analyzed by GC-MS and GC-FID. Shown are GC-FID traces. Peaks of glucosinolate breakdown products are annotated with the retention time and compound abbreviation (nitriles: R-CN; isothiocyanates (blue): R-NCS; side chain abbreviations as in Supplementary Table 5). IS, internal standard. The same principal result was obtained with other individuals from this and other experiments.

**FIGURE 6:**
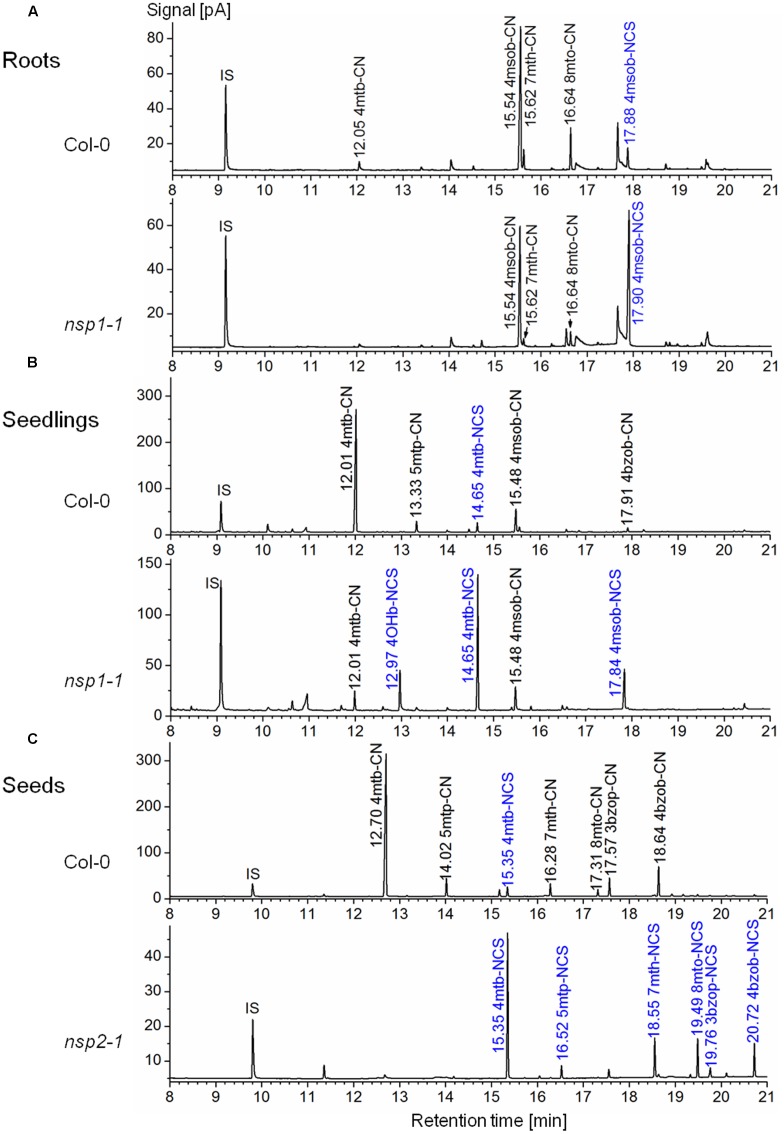
**Glucosinolate breakdown product profiles in homogenates of *A. thaliana* Col-0 WT and mutant plants.** Plant material was homogenized in aqueous buffer and dichloromethane extracts of the homogenates were analyzed by GC-MS and GC-FID. Shown are representative GC-FID traces. Peaks of glucosinolate breakdown products are annotated with the retention time and compound abbreviation (nitriles: R-CN; isothiocyanates (blue): R-NCS; side chains as in Supplementary Table 5). IS, internal standard. **(A)** Roots of individual, 6-week-old aeroponically grown plants. **(B)** Eight-day-old soil-grown seedlings. **(C)** Seeds.

To study the involvement of individual NSPs in glucosinolate breakdown product formation, we prepared aqueous homogenates of fresh plant material from Col-0 WT and mutant plants and analyzed glucosinolate breakdown products in dichloromethane extracts of the homogenates. As expected based on previous research (Lambrix et al., 2001; Burow et al., 2006b), leaf homogenates from individual WT plants contained mixtures of isothiocyanates and simple nitriles derived from the major aliphatic glucosinolates (**Figure 5A**). In contrast, glucosinolate breakdown products in roots of the same individuals were almost exclusively composed of simple nitriles with no or only small amounts of the corresponding isothiocyanates present (**Figure 5B**). Homogenates of WT seeds or seedlings also contained predominantly simple nitriles (**Figures 6B,C**). Breakdown products of indole glucosinolates were detected only rarely and with low abundance, likely due to the fact that indole-3-carbinol derivatives and breakdown products of 1-methoxy- and 4-methoxyindol-3-ylmethylglucosinolate escape GC analysis. Composition of homogenates from mutant plants differed from those of the WT depending on organ and genotype. Namely, simple nitrile levels were reduced in favor of isothiocyanates in affected mutant organs. Examples of changed product profiles in mutants as compared to WT are shown in **Figure 6**.

To analyze the mutant phenotypes in more detail, we attempted to quantify the nitrile to isothiocyanate proportion for the products of the major aliphatic glucosinolates in root, seed and seedling homogenates. However, low recovery of some breakdown products has been observed in a previous study (Witzel et al., 2015). In fact, exact quantification of glucosinolate breakdown products is hampered by high reactivity of the isothiocyanates, chemical instability of some compounds upon GC, and different extractability with dichloromethane leading to variation of recovery. Only some of the breakdown products formed in *A. thaliana* (for example, the isothiocyanates, but not the nitriles, of 4-methylthiobutyl- and 4-methylsulfinylbutylglucosinolate) are commercially available. Thus, recovery of nitriles vs. isothiocyanates cannot be determined experimentally. For a rough estimate of total recoveries, we quantified breakdown products of the major aliphatic glucosinolates in WT root, seedling, and seed homogenates and compared the amount to the content of the precursor glucosinolate (Supplementary Figures 2 and 6). This showed that recovery varied largely and might depend on both analyte structure and sample matrix. Complete recovery of breakdown products relative to glucosinolate content was found only for products of some major glucosinolates, i.e., 4-methylsulfinylbutylglucosinolate in roots (Supplementary Figure 2A), and 4-methylthiobutylglucosinolate in seedlings (Supplementary Figure 2B). In seeds, the total amount of recovered glucosinolate breakdown products varied largely between different seed batches, i.e., seeds of independently grown sets of plants (Supplementary Figure 6). In some batches, levels of breakdown products were in the expected range based on glucosinolate levels, in others they were below the expected range (Supplementary Figure 6) or hardly detectable (data not shown). To test by HPLC analysis if seed homogenates still contained intact glucosinolates, we passed homogenates prepared as above over DEAE-Sephadex A25 columns as for glucosinolate analysis and converted bound glucosinolates to the desulfoderivatives. It turned out that samples with low breakdown product levels had high levels of intact glucosinolates and *vice versa*. For the major glucosinolate 4-methylthiobutylglucosinolate, the amount of breakdown products and intact glucosinolate in the homogenate added up to about the expected range based on glucosinolate levels in intact seeds (Supplementary Figure 6). Taken together, we found large batch-to-batch variation of the extent of glucosinolate breakdown in seed homogenates, but the breakdown products formed were completely recovered for the major glucosinolate 4-methylthiobutylglucosinolate. The glucosinolate breakdown products for which complete recovery was proven in homogenates relative to glucosinolate content in roots, seedlings, and seeds were selected for quantitative assessment of the effects of the mutations on glucosinolate breakdown.

**FIGURE 7:**
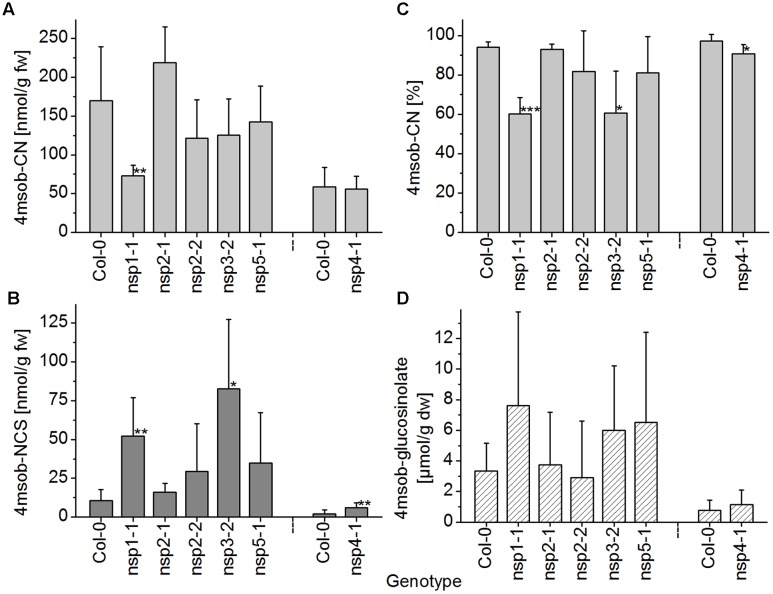
**Breakdown of 4-methylsulfinylbutylglucosinolate in homogenates of roots of WT and *nsp1–nsp5* mutant plants.** Roots of individual, 6-week-old aeroponically grown plants were harvested. One individual was either used for glucosinolate breakdown product quantification by GC-FID of dichloromethane extracts of homogenates of fresh material **(A–C)**, or for determination of glucosinolate content in lyophilized material by HPLC after conversion to the desulfoderivatives **(D)**. **(A)** Absolute amount of nitrile relative to root fresh weight. **(B)** Absolute amount of isothiocyanate relative to root fresh weight. **(C)** Percentage of nitrile relative to the total amount (nmol) of detected breakdown products of 4-methylsulfinylbutylglucosinolate (nitrile+isothiocyanate). **(D)** Root content of 4-methylsulfinylbutylglucosinolate relative to dry weight. Means ± SD of *N* = 6 individuals from two independent experiments (three individuals per experiment) are shown with the exception of the Col-0–*nsp4-1* comparison which was accomplished in a separate set of three independent experiments with three individuals each (*N* = 9). Asterisks denote significant differences to WT (^∗∗∗^*p* < 0.001; ^∗∗^*p* < 0.01; ^∗^*p* < 0.05).

When absolute levels of the nitrile of 4-methylsulfinylbutylglucosinolate in WT and *nsp1–nsp5* root homogenates were compared, only *nsp1-1* had significantly lower levels than the WT (**Figure 7A**). However, although no significant differences in glucosinolate levels between WT and mutant roots were detectable, glucosinolate levels varied largely in roots of different individuals (**Figure 7D**). Thus, decreased nitrile levels could be a consequence of lower glucosinolate levels. To take this into account, we assessed the proportion of nitrile formed relative to the total amount of detected breakdown products. In this comparison, *nsp1-1*, *nsp3-2*, and *nsp4-1* had significantly lower proportions of nitrile than the WT while the other mutants were not affected (**Figure 7C**). This was in agreement with a comparison of absolute isothiocyanate levels between WT and mutant root homogenates. Only homogenates of *nsp1-1*, *nsp3-2*, and *nsp4-1* roots had significantly higher isothiocyanate levels than homogenates of WT plants (**Figure 7B**). The changes in proportion of nitrile formed and the reduced or lacking transcripts in the mutants suggest that 70–80% of the nitrile-forming activity upon breakdown of 4-methylsulfinylbutylglucosinolate in roots is due to an about equal contribution from *NSP1* and *NSP3*. Though *NSP4* contributes to some extent, the relative contribution of *NSP4* and a possible involvement of *NSP5* cannot be evaluated as they are down-regulated, but not knocked-out in the *nsp4-1* and *nsp5-1* mutants, respectively.

**FIGURE 8:**
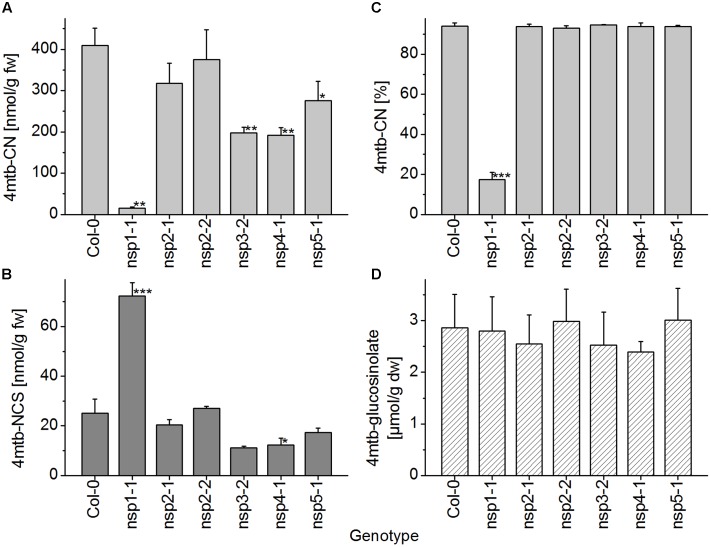
**Breakdown of 4-methylthiobutylglucosinolate in homogenates of WT and *nsp1–nsp5* seedlings.** Batches of 8-day-old soil-grown seedlings were used for glucosinolate breakdown product quantification by GC-FID of dichloromethane extracts of homogenates of fresh material **(A–C)**, or for determination of glucosinolate content in lyophilized material by HPLC after conversion to the desulfoderivatives **(D)**. **(A)** Absolute amount of nitrile relative to seedling fresh weight. **(B)** Absolute amount of isothiocyanate relative to seedling fresh weight. **(C)** Percentage of nitrile relative to the total amount (nmol) of detected breakdown products of 4-methylthiobutylglucosinolate (nitrile+isothiocyanate). **(D)** Seedling content of 4-methylthiobutylglucosinolate relative to dry weight. Means ± SD of *N* = 3 independently grown batches of seedlings. Asterisks denote significant differences to WT (^∗∗∗^*p* < 0.001; ^∗∗^*p* < 0.01; ^∗^*p* < 0.05).

In seedling homogenates, the absolute level of the nitrile derived from 4-methylthiobutylglucosinolate was significantly reduced in the *nsp1-1*, *nsp3-2*, *nsp4-1*, and *nsp5-1* mutants (**Figure 8A**). However, the total level of the corresponding isothiocyanate was significantly increased relative to WT only in the *nsp1-1* mutant (**Figure 8B**). In agreement with this observation, the proportion of nitrile to isothiocyanate derived from 4-methylthiobutylglucosinolate was affected only in the *nsp1-1* mutant (**Figure 8C**). Nitrile formation was reduced from 94% of total products in WT to 17% in *nsp1-1*. Levels of 4-methylthiobutylglucosinolate did not differ significantly between WT and mutant seedlings (**Figure 8D**). Thus, more than 80% of the nitrile-forming activity upon breakdown of 4-methylthiobutylglucosinolate in seedlings seems to be due to *NSP1*. Whether *NSP3*, *NSP4*, and *NSP5* also contribute to nitrile formation remains unclear as the observed decrease in nitrile formation was not accompanied by a significant increase in isothiocyanate levels in the respective mutants. Thus, it could also be due to decreased glucosinolate breakdown (i.e., lower myrosinase activity).

**FIGURE 9:**
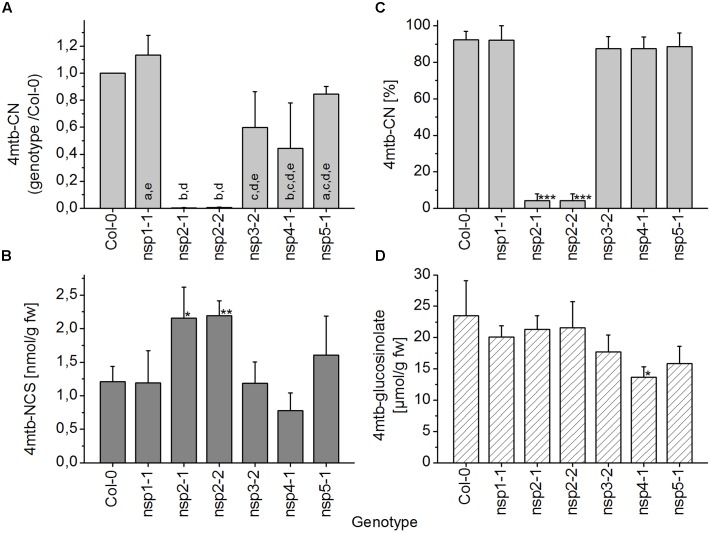
**Breakdown of 4-methylthiobutylglucosinolate in homogenates of WT and *nsp1–nsp5* seeds.** Batches of seeds were used for glucosinolate breakdown product quantification by GC-FID of dichloromethane extracts of homogenates of fresh material **(A–C)**, or for determination of glucosinolate content by HPLC after conversion to the desulfoderivatives **(D)**. **(A)** Absolute amount of nitrile (nmol per g fresh weight) was determined for each genotype and divided by the amount determined for WT. Significant differences between the mutants (*p* < 0.05, ANOVA with Tukey’s Test) are indicated by different letters in the columns. **(B)** Absolute amount of isothiocyanate relative to fresh weight. **(C)** Percentage of nitrile relative to the total amount (nmol) of detected breakdown products of 4-methylthiobutylglucosinolate (nitrile+isothiocyanate). **(D)** Seed content of 4-methylthiobutylglucosinolate. Means ± SD of seeds from *N* = 3 independently grown sets of plants. Asterisks in **(B–D)** denote significant differences to WT (^∗∗∗^*p* < 0.001; ^∗∗^*p* < 0.01; ^∗^*p* < 0.05).

As explained above, the extent of glucosinolate breakdown in WT seed homogenates varied between seeds obtained from independently grown batches of plants, meaning that in some batches a relatively large proportion of seed glucosinolate remained intact upon homogenization in aqueous buffer. This led to variation of absolute nitrile levels between experiments (Supplementary Figure 7). We therefore calculated the ratio between levels in mutants and WT for each experiment before statistical analysis (**Figure 9A**). In all experiments, the nitrile was either detected at very low levels or was undetectable in homogenates of *nsp2-1* and *nsp2-2* seeds while levels of 7–27 μmol/g were found for WT seeds. Seeds of *nsp1-1* produced about the same level of nitrile as the WT. Nitrile levels in seed homogenates of *nsp3-2*, *nsp4-1*, and *nsp5-1* were consistently lower than in those of WT, but subject to large variation. As a possible reason for decreased nitrile levels, the seed content of the precursor glucosinolate, 4-methylthiobutylglucosinolate, was also consistently reduced in *nsp3-2*, *nsp4-1*, and *nsp5-1* relative to WT (**Figure 9D**). But this was significant only for *nsp4-1*. In *nsp2-1* and *nsp2-2* seed homogenates, the percentage of nitrile relative to the total amount of detected breakdown products of 4-methylthiobutylglucosinolate was significantly lower than that of WT seed homogenates (**Figure 9C**). In contrast to nitrile levels, absolute isothiocyanate levels were in the same range in different experiments, so data were directly pooled across experiments. Homogenates of *nsp2-1* and *nsp2-2* mutant seeds contained significantly higher isothiocyanate levels than those of WT seeds (**Figure 9B**). The other mutants did not differ significantly from WT in terms of isothiocyanate formation. Thus, we can conclude that more than 99% of the nitrile-forming activity upon breakdown of 4-methylthiobutylglucosinolate in seeds is due to *NSP2*.

## Discussion

*Arabidopsis thaliana* Col-0 has been regarded as an accession which produces mainly isothiocyanates upon glucosinolate breakdown following tissue disruption (Lambrix et al., 2001; Burow et al., 2006b). While this has been proven experimentally for rosette leaves (Lambrix et al., 2001) and the inflorescence (Falk et al., 2014), our present study demonstrates that simple nitriles are the predominant breakdown products of at least the major aliphatic glucosinolates in roots, seedlings, and seeds of this accession. This observation is in agreement with the expression of one or several *NSP* genes in these organs. Involvement of *NSPs* in simple nitrile formation is further supported by reduced simple nitrile levels in homogenates of mutant lines with T-DNA insertions in *NSP* genes. Although the *nsp4-1* and *nsp5-1* mutants showed reduced target gene expression and changes in breakdown product formation, it remains difficult to evaluate the quantitative contribution of *NSP4* and *NSP5* to nitrile formation. However, our analysis only covered breakdown of major aliphatic glucosinolates and would have missed effects on breakdown of, e.g., indolic glucosinolates. Moreover, post-transcriptional regulation could affect mutant phenotypes as previously proposed (Burow et al., 2009). In contrast, lack of nitrile formation upon homogenization of seeds of two independent knockout lines, *nsp2-1* and *nsp2-2*, establishes a direct link between *NSP2* expression and a high nitrile:isothiocyanate proportion in seed homogenates. Based on microarray data^1^ (Hruz et al., 2008; Supplementary Figure 1), *NSP2* expression is high in siliques, but low in inflorescences and other plant parts, and none of the other *NSP* genes has a pronounced expression in inflorescences and siliques. This is in agreement with our data on seeds and a previous report on high isothiocyanate levels in homogenates of *A. thaliana* Col-0 inflorescences (Falk et al., 2014). Interestingly, *NSP2* expression was not detectable anymore in 8-day-old seedlings, even though they also produced high simple nitrile levels upon homogenization. This indicates a specific role of *NSP2* in seeds. As a possible scenario, isothiocyanate defense might only be desirable for the plant as long as flowers develop. Upon seed maturation consumption of siliques by herbivores could support seed dispersal. In fact, larvae of the generalist lepidopteran herbivore *Trichoplusia ni* (Noctuidae) avoided feeding on Col-0 inflorescences (Jander et al., 2001). However, the generalist slug *Arion lusitanicus* (Arionidae) did not discriminate between inflorescences of Col-0 and transgenics with increased proportion of nitriles formed upon damage (Falk et al., 2014). To get a better understanding of glucosinolate breakdown and its roles in generative organs, future studies should address breakdown product profiles and *NSP1–NSP5* expression during flower, silique, and seed development. The *nsp2-1* and *nsp2-2* mutant lines could be used to test if nitrile vs. isothiocyanate formation affects herbivore feeding on siliques. We have presently no explanation for the low extent of glucosinolate breakdown in homogenates of some seed batches. Increased incubation times before dichloromethane extraction did not increase breakdown product levels, and contents of breakdown products and intact glucosinolates in homogenates did not generally depend on seed storage time after harvest within a time frame of 7 months (data not shown). Expression, activity and possible inhibition of myrosinases in the seed remain a subject for future studies.

From day 2 to day 8 of seed-to-seedling development of *A. thaliana*, total glucosinolate content per individual decreases by 30% indicating turnover of glucosinolates (Brown et al., 2003). The mechanism of glucosinolate turnover has not been elucidated yet, but a pathway has been proposed in which thioglucosidase(s), NSP(s), and nitrilase(s) cooperate to produce a carboxylic acid under release of sulfur and ammonia besides glucose and sulfate (**Figure 1C**) (Janowitz et al., 2009). This pathway would not only help to avoid formation of reactive isothiocyanates, but contribute nutrients for plant growth and development. A defect in the *NSPs* could be expected to disturb this pathway and to negatively affect germination and/or growth. Although we did not assess germination and seedling growth systematically, we did not observe any obvious signs of impaired seedling development of the analyzed mutants, maybe due to functional redundancy of *NSPs* in embryos and early seedling stages or due to use of fertilized soil. In 8-day-old seedlings, the *nsp1-1* allele caused the strongest reduction of simple nitrile formation among the genotypes tested, but simple nitrile formation was also reduced in other mutant lines relative to WT. More detailed studies on *NSP* expression in seed, embryo and seedling are needed to clarify in which tissues *NSP2* is expressed and at which stage *NSP2* expression is superseded by expression of other *NSPs*. Mutants with T-DNA insertions in multiple *NSPs* would be helpful to dissect a possible role of *NSPs* in seedling development and glucosinolate catabolism. However, only some of the desirable mutants can be generated by crossing as *NSP1*, *NSP3*, and *NSP4* are tandem genes. Mutants of multiple *NSPs* would also be valuable tools to study roles of simple nitrile formation in roots. Based on our analyses, *NSP1* and *NSP3* are the major contributors to simple nitrile formation in roots. As long as double mutants are not available, the *nsp1-1* mutant with the most consistent breakdown product phenotype could be used in comparison to WT to study roles of simple nitriles in interactions of the plant with root herbivores and soil microbes. As above-ground and below-ground organs of *A. thaliana* express distinct myrosinase isoforms (Andersson et al., 2009), future studies should consider the organ-specific combination of NSPs and myrosinases and test its physiological and ecological relevance. NSP involvement in glucosinolate breakdown by atypical myrosinases such as the root-expressed PYK10 (Nakano et al., 2016) will also be a promising field of future research. Given the problem of low recovery can be overcome, it would be very interesting to extend this analysis to breakdown of other aliphatic and major indolic glucosinolates to assess whether NSP1-NSP5 activity differentially affects breakdown of structurally diverse glucosinolates.

Previous research addressed changes of glucosinolate breakdown product profiles upon *Verticillium longisporum* (Ascomycota) infection of four *A. thaliana* accessions with alkenyl-, hydroxyalkenyl-, hydroxyalkyl-, and/or methylsulfinylalkylglucosinolates as major aliphatic glucosinolates and found accession-specific alterations of product types and total quantities (Witzel et al., 2015). Results obtained from uninfected control plants were, in part, similar to our results. For example, ESP-independent simple nitrile formation dominated over isothiocyanate formation in root homogenates of the accessions Hilversum (Hi-0) and Burren (Bur-0) (Witzel et al., 2015). An involvement of *NSP* genes was, however, not investigated. In agreement with this, allyl- or benzylglucosinolate added to homogenates of roots of a range of *A. thaliana* accessions were hydrolyzed mainly to the simple nitrile regardless of a low nitrile proportion in homogenates of above-ground plant organs (Wentzell and Kliebenstein, 2008). Thus, simple nitrile formation in roots upon homogenization might be a very common phenomenon in *A. thaliana*.

It would be interesting to include more accessions and other species of the Brassicaceae into a quantitative survey of glucosinolate breakdown products formed in homogenates of different organs. A screening of species from nine genera of the Brassicaceae for formation of alternative (non-isothiocyanate) breakdown products included different organs and showed that formation of alternative breakdown products is quite widespread within the Brassicaceae (Kuchernig et al., 2012). Simple nitriles have also been identified as major breakdown products released from green manures into soil (Gardiner et al., 1999). In this case, simple nitrile formation could be due to NSP activity and/or due to the specific reaction conditions (pH, iron content) in the soil (Borek et al., 1994). Information on breakdown product types and their proportions might be essential for proper interpretation of results obtained in bioassays. Breakdown product analysis should best be conducted with fresh plant material as specifier protein-mediated product formation is sensitive to freezing. The use of frozen plant material would therefore bear the risk of overlooking formation of alternative breakdown products such as simple nitriles.

## Conclusion

Our study demonstrates predominant formation of simple nitriles upon hydrolysis of major endogenous aliphatic glucosinolates of roots, seedlings, and seeds of *A. thaliana* Col-0. Supported by glucosinolate breakdown product profiles of two independent T-DNA insertion lines, simple nitrile formation in seed homogenates is solely due to *NSP2*. Our analyses further suggest that *NSP1* controls simple nitrile formation in homogenates of seedlings with minor contributions from other *NSP* genes, while breakdown product profiles of roots depend mostly on *NSP1* and *NSP3*. Thus, evolution of *NSP* genes in *A. thaliana* has been associated with changes in organ-specific regulation of gene expression. As many biological activities of glucosinolates are attributed to their breakdown products, glucosinolate breakdown product profiles and their variation within a plant deserve attention in future research on the ecological roles of the glucosinolate-myrosinase system.

## Author Contributions

UW designed the study, KM, FD, and BMR conducted the experiments, UW, KM, FD, and BMR analyzed the data; UW drafted the manuscript, KM, FD, and BMR revised the manuscript; all authors approved the final version of the manuscript and agreed to be accountable for the content of the work.

## Conflict of Interest Statement

The authors declare that the research was conducted in the absence of any commercial or financial relationships that could be construed as a potential conflict of interest.

## Footnotes

1 www.genevestigator.com
