# Supplementary material for: NSP-Dependent Simple Nitrile Formation Dominates upon Breakdown of Major Aliphatic Glucosinolates in Roots, Seeds, and Seedlings of Arabidopsis thaliana Columbia-0
Source: Front Plant Sci. 2016 Dec 1;7:1821. doi: 10.3389/fpls.2016.01821 (PMC5131009; doi:10.3389/fpls.2016.01821)
Supplement: Supplementary file 1 [file Presentation_1.PDF]

Supplementary Material

NSP-dependent simple nitrile formation dominates upon breakdown of major aliphatic glucosinolates in roots, seeds, and seedlings of *Arabidopsis thaliana* Columbia-0

Running title: Nitrile formation in Arabidopsis

Ute Wittstock[1,*], Kathrin Meier[1], Friederike Dörr[1], Beena M. Ravindran[1,#]

*** Correspondence:** Ute Wittstock, u.wittstock@tu-bs.de

1 Supplementary Figures and Tables

1.1 Supplementary Figures

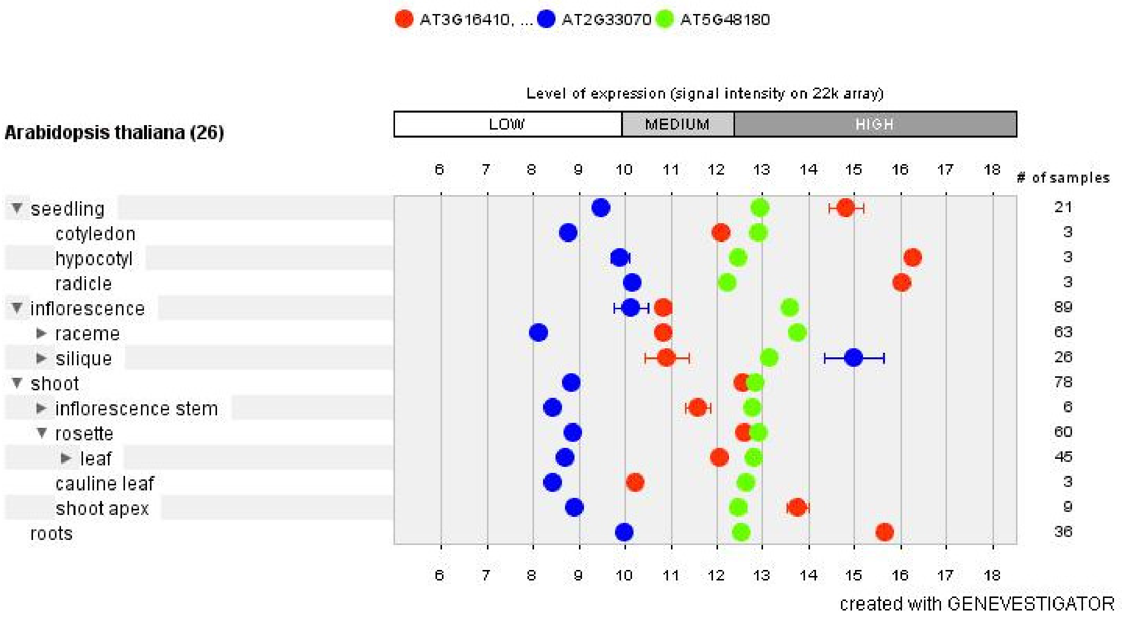

Supplementary Figure 1. Expression analysis of NSP1-NSP5 by GENEVESTIGATOR (Hruz *et al.*, 2008). Individual experiments on Arabidopsis development and organ-specific expression were selected (AT-00087, AT-00088, AT-00089, AT-00091, AT-00092, AT-00093, AT-00121, AT-00490) and wildtype microarray data (ATH1:22k array) analyzed for probes 259381_s_at (NSP1, NSP3, NSP4, red), 245161_at (NSP2, blue), and 248713_at (NSP5, green). The figure was generated by GENEVESTIGATOR.

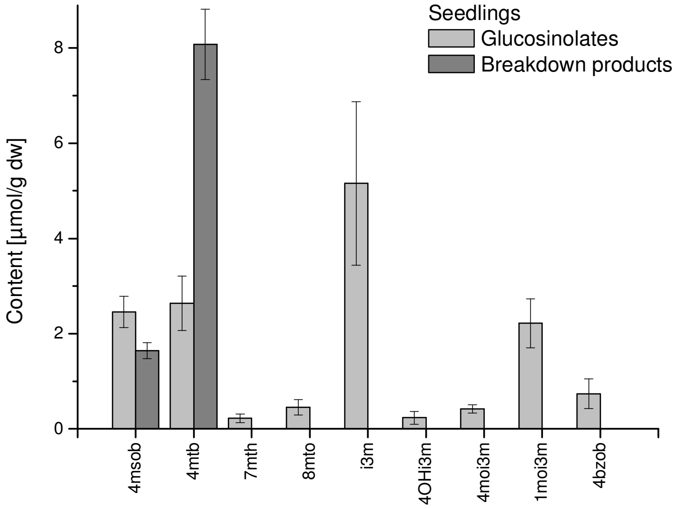

A

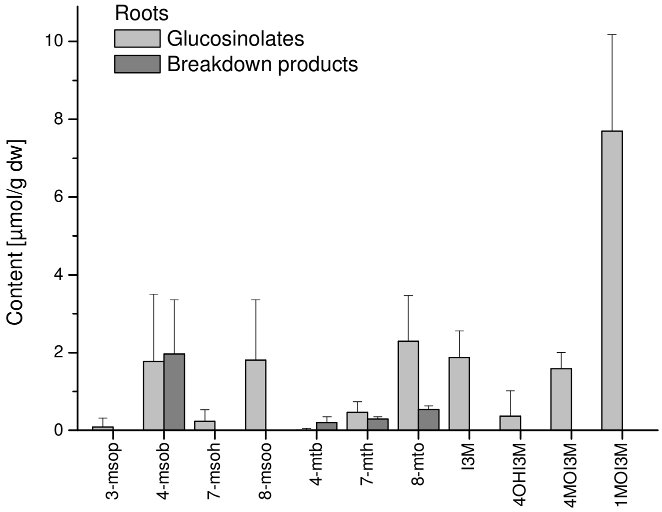

B

Supplementary Figure 2. Glucosinolate content in roots and seedlings of *A. thaliana* Col-0 plants and recovery of breakdown products. Roots of individual six-week-old aeroponically grown plants (**A**) and batches of eight-day-old soil-grown seedlings (**B**) were either freeze-dried for glucosinolate determination by HPLC or homogenized directly after harvest for quantification of glucosinolate breakdown products by GC-FID. Breakdown product content per g dry weight was calculated based on experimentally determined factors for fresh weight-dry weight conversion. A: Data are from five independent experiments with one to three individuals each (total of 13-15 individuals). Means ± SD (N=13-15) are given. B: Data are from six batches of seedlings. Means ± SD (N=6) are given. Side chain abbreviations as in Table S5.

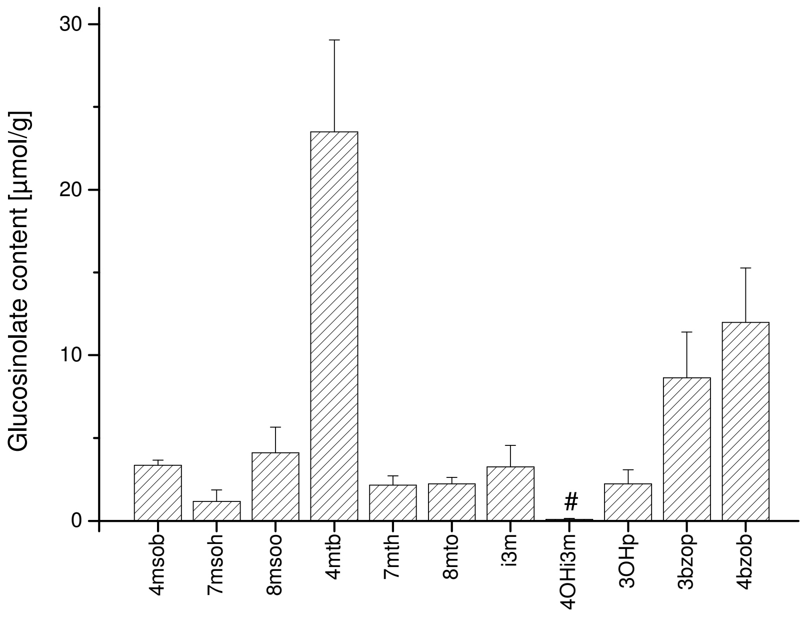

Supplementary Figure 3. Glucosinolate content in seeds of *A. thaliana* Col-0 plants. Seeds were harvested upon maturation of siliques and stored for one week before extraction. Glucosinolates were quantified by HPLC after conversion to the desulfo-derivatives. Means ± SD of seeds of N=3 independently grown sets of plants (#, Mean <0.1 µmol/g). Side chain abbreviations as in Table S5.

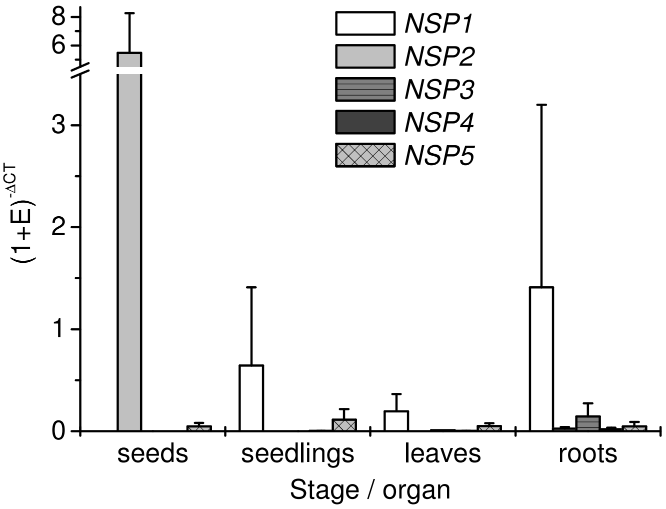

Supplementary Figure 4. qPCR analysis of *NSP1-NSP5* expression in *A. thaliana* Col-0 with *UBI10* as reference gene. Results are shown for seeds, eight-day-old, soil-grown seedlings, as well as rosettes and roots of six-week-old, aeroponically grown plants. qPCR analysis was done using SYBR Green for quantification. The CT values of *NSP1-NSP5* were normalized to *UBI10* as reference (Ref) gene. The expression level of the gene of interest (GOI) in each tissue was expressed as $(1+E)^{-\Delta CT} = (1+E_{Ref})^{CTRef} / (1+E_{GOI})^{CTGOI}$ (E, primer efficiency). Means ± SD of n=3 independently grown sets of plants.

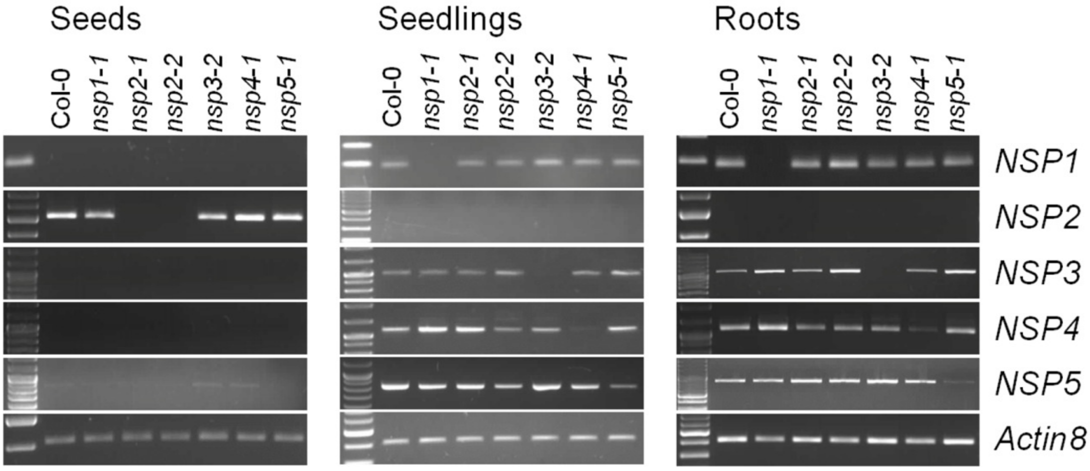

Supplementary Figure 5. Semiquantitative RT-PCR analysis of *NSP1-NSP5* expression in T-DNA insertion lines of *A. thaliana*. Results are shown for seeds, eight-day-old, soil-grown seedlings, and roots of six-week-old, aeroponically grown plants. Total RNA (0.25 µg) was used for cDNA synthesis, and cDNA was subjected to PCR with the gene-specific primers indicated at the right. Gel images are from one experiment that was repeated once with an independently grown set of plants and gave the same principal results.

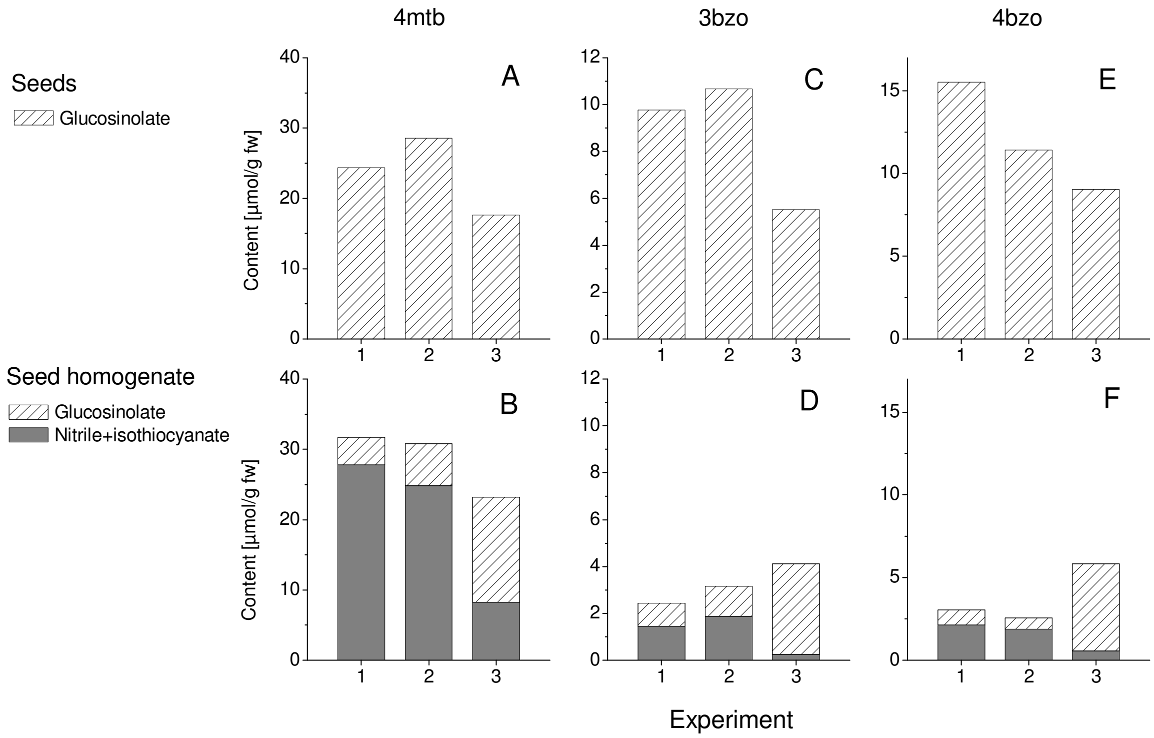

Supplementary Figure 6. Recovery of glucosinolate breakdown products relative to glucosinolate content in seeds of *A. thaliana* Col-0 plants. Seeds were harvested upon maturation of siliques and stored for one week before extraction. Experiments 1-3 were done with seeds from independently grown sets of plants. (**A**, **C**, **E**) Glucosinolates were quantified by HPLC after conversion to the desulfo-derivatives. (**B**, **D**, **F**). Aqueous homogenates of seeds were analyzed for glucosinolate breakdown products by GC-MS and GC-FID and for remaining intact glucosinolates by HPLC after conversion to the desulfo-derivatives ((**A**, **B**): 4-methylthiobutylglucosinolate /breakdown products; (**C**, **D**): 3-benzoyloxypropylglucosinolate /breakdown products; (**E**, **F**): 4-benzoyloxybutylglucosinolate /breakdown products). Side chain abbreviations as in Table S5.

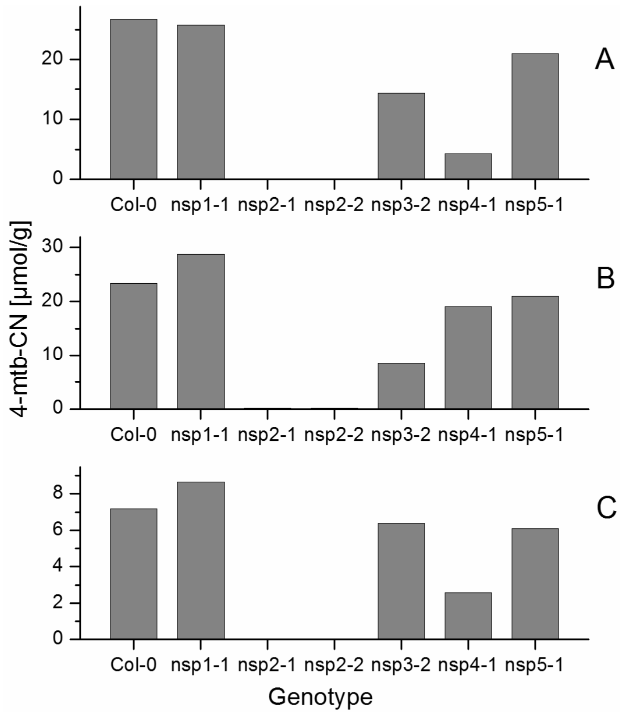

Supplementary Figure 7. Breakdown of 4-methylthiobutylglucosinolate in homogenates of wildtype and *nsp1-nsp5* seeds. Batches of seeds were used for glucosinolate breakdown product quantification by GC-FID of dichloromethane extracts of homogenates of fresh material. Absolute amount of nitrile (nmol per g fresh weight) is shown as determined for seeds of three independently grown sets of plants (**A-C**). Side chain abbreviations as in Table S5.

1.2 Supplementary Tables

Supplementary Table 1. Confirmation of T-DNA insertions in the *NSP1-NSP5* genes. Primer combinations were used for PCR on genomic DNA isolated from Col-0 and mutant plants. PCR products were cloned and sequenced.

AGI code	Allele (Mutant stock name)	Primer combination	Product identity
At3g16400	*NSP1*	KO1F+KO1R<br>KO1F+LB<br>LB+KO1R	578 bp *NSP1*<br>-<br>-
	nsp1-1 (SALK_072600)	KO1F+KO1R<br>KO1F+LB<br>LB+KO1R	-<br>522 bp *NSP1*+ T-DNA<br>-
At2g33070	*NSP2*	KO2F+KO2R<br>LB+KO2R	681 bp *NSP2*<br>-
	nsp2-1 (SALK_004170-3)	KO2F+KO2R<br>LB+KO2R	-<br>551 bp *NSP2* + T-DNA
	nsp2-2 (SALK_057194-1)	KO2F+KO2R<br>LB+KO2R	-<br>461 bp *NSP2* + T-DNA
At3g16390	*NSP3*	KO3F3+KO3R2<br>KO3F3+LB<br>LB+KO3R2	358 bp *NSP3*<br>-<br>-
	nsp3-2 (SALK_016880-4)	KO3F3+KO3R2<br>KO3F3+LB<br>LB+KO3R2	-<br>71 bp *NSP3* + T-DNA<br>321 bp *NSP3* + T-DNA
At3g16410	*NSP4*	KO4F+KO4R<br>KO4F+LB3	933 bp *NSP4*<br>-

	nsp4-1 (SAIL_904_E01)	KO4F+KO4R<br>KO4F+LB3	-<br>776 bp *NSP4* + T-DNA
At5g48180	*NSP5*	KO5F+KO5R<br>KO5F+LB	785 bp *NSP5*<br>-
	nsp5-1 (SALK_121606-1)	KO5F+KO5R<br>KO5F+LB	-<br>618 bp *NSP5* + T-DNA

Supplementary Table 2. Primers used for semi-quantitative RT-PCR.

Gene	Primer	Primer sequence (5'-3')	Annealing temperature	Product size (cDNA; bp)
NSP1	RT1F	ATGTAGGGCAAGCACAATAC	63°C	193
	RT1R	TCATTTCTGACGTCATATCGT		
NSP2	RT2F	CTATATATCATCGCCGGCCACTC	65°C	514
	RT2R	CGAACACTGCACCCACTTGTG		
NSP3	RT3F	GCCTATCGTTAAAAAACCTGGGG	65°C	961
	RT3R	CTACTCGGAGTCCCCTCAAACTTATC		
NSP4	RT4F	GATCCGAATACGACGCTGTGAC	63°C	1044
	RT4R	AGAGGGTTGGAATGTCTCCC		
NSP5	RT5F	GAGAACAAATGGTTAAAGGTGGG	65°C	802
	RT5R	CAATCCTCTCCCACACTAACGTCTC		
Act8	Actin8F	AGCTGTTCTATCACTTTACGCCAG	63°C	412
	Actin8R	GATCCCTGCAGCTTCCATCC		

Supplementary Table 3. Primers used for qPCR. Primer efficiencies (E) for *NSP1*, *NSP4*, and *NSP5* were determined as means of independent runs with cDNA obtained from seedlings, rosettes and roots (n=3; relative SD <5 %). Primer efficiencies for *EF-1α* and *UBI10* were determined as means of independent runs with cDNA obtained from seeds, seedlings, rosettes and roots (n=4; relative SD <5 %). Primer efficiencies for *NSP2* and *NSP3* were determined with cDNA from seeds and roots, respectively.

Gene	Primer	Primer sequence (5'-3')	Product size (cDNA; bp)	1+E
NSP1	q1F1	GGGCAAGCACAATACGGTATAGC	192	1.96
	q1R2a	CGTGATCATTTCTGACGTCATATCGT		
NSP2	RT2F	CTATATATCATCGCCGGCCACTC	143	1.93
	q2R3	GTGTGAGCTCACCACCAAATGAG		
NSP3	q3F1	CTCAGCCTATCGTTAAAAAACCTGGGG	168	1.96
	q3R1	CTCCATTTTGCTCCACCTTAATCCAG		
NSP4	RT4F	GATCCGAATACGACGCTGTGAC	180	2.04
	q4R2	AAACTAAGTGCTCCTCCGGATG		
NSP5	q5F1	GGTGGAGAACAAATGGTTAAAGGTGG	139	2.00
	q5R3	ATCGTTATCAATATGGATCGTTGGCTTAAG		
EF-1α	qEF-F1	AGGCTGGTATCTCTAAGGATGGTCAG	107	1.98
	qEF-R	GGTGGTGGCATCCATCTTGTTACA		
UBI10	qUBIF2	AACTTTCTCTCAATTCTCTCTACCGTGATC	90	1.93
	qUBIR7	GCTTTCCACCTCGAGGGTGATT		

TUB6	qb6TF1	GTATGGTTTTGGACAATGAAGCCCT	143	2.00
	qb6TR1	GAGCTGACCCGGGAACCTAA		

Supplementary Table 4. Primers used for confirmation of mutant alleles by PCR.

Primer abbreviation	Primer long name	Sequence (5'-3')
LB	pROK2LB2	CGGGACCGCTTGCTGC
LB3	LB3.R	TTCATAACCAATCTCGATACAC
KO1F	At3g16400_fw1	TTTGAGATTGATGCGGATGACTAC
KO1R	At3g16400_rev1	CCTACTTGTGCTATGGGATGTGAG
KO2F	At2g33070_fw1	ATTTTTCTCTTCTATTCAATACGATTATCTTAG
KO2R	AT2G33070_REV1	CTTTCTCATGATAACCTTCCACGTATAC
KO3F3	KO3F3	GGTATATGTAGGGCAAGGCCAG
KO3R2	KO3R2	CCCCAGGTTTTTTAACGATAGGC
KO4F	nsp4-1.F	CAGCCACGGGAGACATTCCAAC
KO4R	nsp4-1.R	GAAACATAAAACAATTAAACATATAG
KO5F	AT5G48180_FW1	GCTGGTGATGATCGGAAAGTTTATG
KO5R	AT5G48180_Rv1	TCATACTTCAATAATTTCGTTCTTTG

Supplementary Table 5. Glucosinolate side-chain abbreviations used in figures.

**Abbreviation**	**Side chain**
4mtb	4-methylthiobutyl-
5mtp	5-methylthiopentyl-
7mth	7-methylthioheptyl-
8mto	8-methylthiooctyl-
3msop	3-methylsulfinylpropyl-
4msob	4-methylsulfinylbutyl-
7msoh	7-methylsulfinylheptyl-
8msoo	8-methylsulfinyloctyl-
4OHb	4-hydroxybutyl-
3bzop	3-benzoyloxypropyl-
4bzob	4-benzoyloxybutyl-
i3m	indol-3-ylmethyl-
4OHi3m	4-hydroxyindol-3-ylmethyl-
4moi3m	4-methoxyindol-3-ylmethyl-
1moi3m	1-methoxyindol-3-ylmethyl-
